# Characterization of a critically ill population with legionella pneumonia during a severe community outbreak in portugal

**DOI:** 10.1186/2197-425X-3-S1-A354

**Published:** 2015-10-01

**Authors:** J Ribeiro, J Nave, C Candeias, R Marques, R Marcal, C Santos, J Monteiro, I Moniz, Z Costa e Silva, C Franca

**Affiliations:** Intensive Care Department, University Hospital of Santa Maria, Lisbon, Portugal; Infectious Diseases Department, University Hospital Santa Maria, Lisbon, Portugal; Pulmonology Department, University Hospital Santa Maria, Lisbon, Portugal

## Introduction

A major community outbreak of Legionnaire' disease occurred in the great region of Lisbon, from October to December 2014. This outbreak resulted in a large number of patients developing severe pneumonia and demanding treatment in intensive care units (ICU). Information regarding this specific population of patients with severe Legionella pneumonia is very scarce in the literature.

## Objectives

Clinical, functional, laboratorial, and prognostic characterization of the population of patients with the most severe forms of Legionella pneumonia treated in an ICU.

## Methods

Prospective registration and statistical analysis of data based on a protocol specifically designed for the population of 24 patients with Legionella pneumonia admitted to ICU.

## Results

From October to December a community outbreak of Legionnaire's disease infected 375 patients. Sequence-based typing of clinical and environmental isolates confirmed industrial wet cooling systems as the source of infection. Seventy-six patients were treated at our hospital, of which twenty-four demanded ICU admission, mainly due to severe hypoxemic respiratory failure. Median age were 56.9 ± 11.9 (20-77), with a preponderance of male sex (17 vs. 7). SAPS II score was 41.25 ± 16.61, SOFA at admission was 6.7 ± 3.1 and mean number of failing organs was 2.8 ± 1.4. Sixteen patients (66.7%) demanded invasive mechanical ventilation, and 6 patients had to be rescued by extracorporeal membrane oxygenation (median time of ECMO run of 7.5 ± 2.3). ICU and hospital length of stay were 14.3 ± 4.7 and 25.6 ± 14.0, respectively, and hospital mortality was 8,3% (2 patients). Patients were treated with azithromycin (17) or levofloxacin (7 patients). A moderate correlation was identified between the mean time to a 50% value reduction of C-reactive protein and hospital LOS; degree of hypoxemia at admission (Pa02:Fi02 147.2 ± 86.8) moderately predicted the need for ECMO; SAPS II and C-reactive protein value at admission (45.8 ± 39.9 mg/dl) did not correlate with LOS (ICU or hospital) nor with the need for ECMO.

## Conclusions

The authors characterize a population of patients with Legionella pneumonia that needed an ICU-based treatment approach. To the best of their knowledge, this is one of the biggest series of patients with a diagnosis of Legionella pneumonia that evolved to the most severe forms of the disease and should contribute to a better understanding of this entity.

## Grant Acknowledgment

No grant disclosures.
